# Delayed Diagnosis of a Ruptured Distal Posterior Inferior Cerebellar Artery (PICA) Dissecting Aneurysm Presenting as Craniocervical Junction and Spinal Subdural Hematoma

**DOI:** 10.7759/cureus.71830

**Published:** 2024-10-19

**Authors:** Yuma Hiratsuka, Koichiro Shindo, Yasufumi Ohtake, Hirohiko Nakamura

**Affiliations:** 1 Department of Neurosurgery, Nakamura Memorial Hospital, Sapporo, JPN

**Keywords:** craniocervical junction, dissecting aneurysm, posterior inferior cerebellar artery, spinal subdural hematoma, subarachnoid hemorrhage

## Abstract

Posterior inferior cerebellar artery (PICA) dissecting aneurysms are rare and typically present with subarachnoid hemorrhage (SAH) or ischemic symptoms, with a high risk of rebleeding in the acute phase. This case presents an atypical ruptured PICA aneurysm with a hematoma confined to the craniocervical junction and cervical cord, leading to a delayed diagnosis - a 41-year-old male with an atypical presentation of headache and neck pain without neurological deficits. Initial magnetic resonance imaging (MRI) revealed a hematoma extending from the craniocervical junction to the cervical spinal cord without intracranial SAH, leading to misdiagnosis as spinal subdural hematoma. As symptoms worsened, further investigation with MRI and digital subtraction angiography (DSA) uncovered a ruptured PICA-dissecting aneurysm. The patient underwent successful endovascular coil embolization with parent artery occlusion. This case highlights the importance of considering PICA-dissecting aneurysms in craniocervical junctions and spinal cord hemorrhage, even in the absence of typical intracranial SAH. It underscores the need for a high index of suspicion and comprehensive vascular imaging for timely diagnosis and treatment in atypical cases.

## Introduction

Aneurysms of the posterior inferior cerebellar artery (PICA) account for 1.4%-4.5% of all intracranial aneurysms and can occur in any segment of the PICA [1]. Among these, PICA-dissecting aneurysms are particularly uncommon, comprising only 0.5%-0.7% of all intracranial aneurysms [2]. Diagnosis of PICA aneurysms typically involves digital subtraction angiography (DSA), CT angiography, or MR angiography. However, misdiagnosis is not uncommon due to the thin lumen of the PICA, which can make accurate identification challenging [3,4]. These aneurysms typically present with subarachnoid hemorrhage (SAH) or ischemic symptoms due to dissection. PICA-dissecting aneurysms carry a high risk of rebleeding in the acute phase, with a rate of approximately 24% and an associated high mortality [5]. Therefore, a prompt diagnosis and therapeutic intervention are crucial for improving patient outcomes.

While PICA-dissecting aneurysms typically present with intracranial subarachnoid hemorrhage, they can occasionally manifest with atypical bleeding patterns. In rare cases, the bleeding can extend into the spinal canal, mimicking a spinal hemorrhage [6]. Conversely, spontaneous spinal hematoma is itself an uncommon condition, with many cases having no clear etiology [7,8]. The similarity in presentation between spontaneous spinal hematomas and atypical PICA aneurysm ruptures can pose significant diagnostic challenges, potentially leading to delayed recognition of the underlying vascular pathology.

Here, we present a case of a ruptured dissecting aneurysm in the distal segment of the PICA that initially presented as a hematoma localized to the craniocervical junction and cervical cord, without apparent intracranial SAH. This atypical presentation led to a delayed diagnosis. We report this case to highlight the diagnostic challenges associated with unusual presentations of PICA-dissecting aneurysms.

## Case presentation

**Figure 1:**
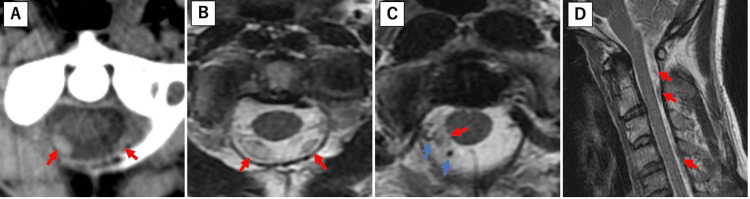
Hematomas (red arrows) are observed at the craniocervical junction of the cervical spine. Flow voids (blue arrows) are observed around the hematoma at the craniocervical junction. (A) CT axial, (B, C) T2WI axial, and (D) T2WI sagittal. T2WI: T2-weighted imaging.

A 41-year-old man presented to a local neurosurgical hospital with complaints of headache and posterior neck pain. His medical history was significant only for well-controlled hypertension. On the initial examination, the patient was alert and oriented. Neurological examination revealed no focal deficits or abnormalities. Magnetic resonance imaging (MRI) was performed, and T2-weighted images revealed a hematoma extending from the craniocervical junction to the cervical spinal cord. Flow voids were observed around the hematoma at the craniocervical junction (Figure 1).

**Figure 2:**
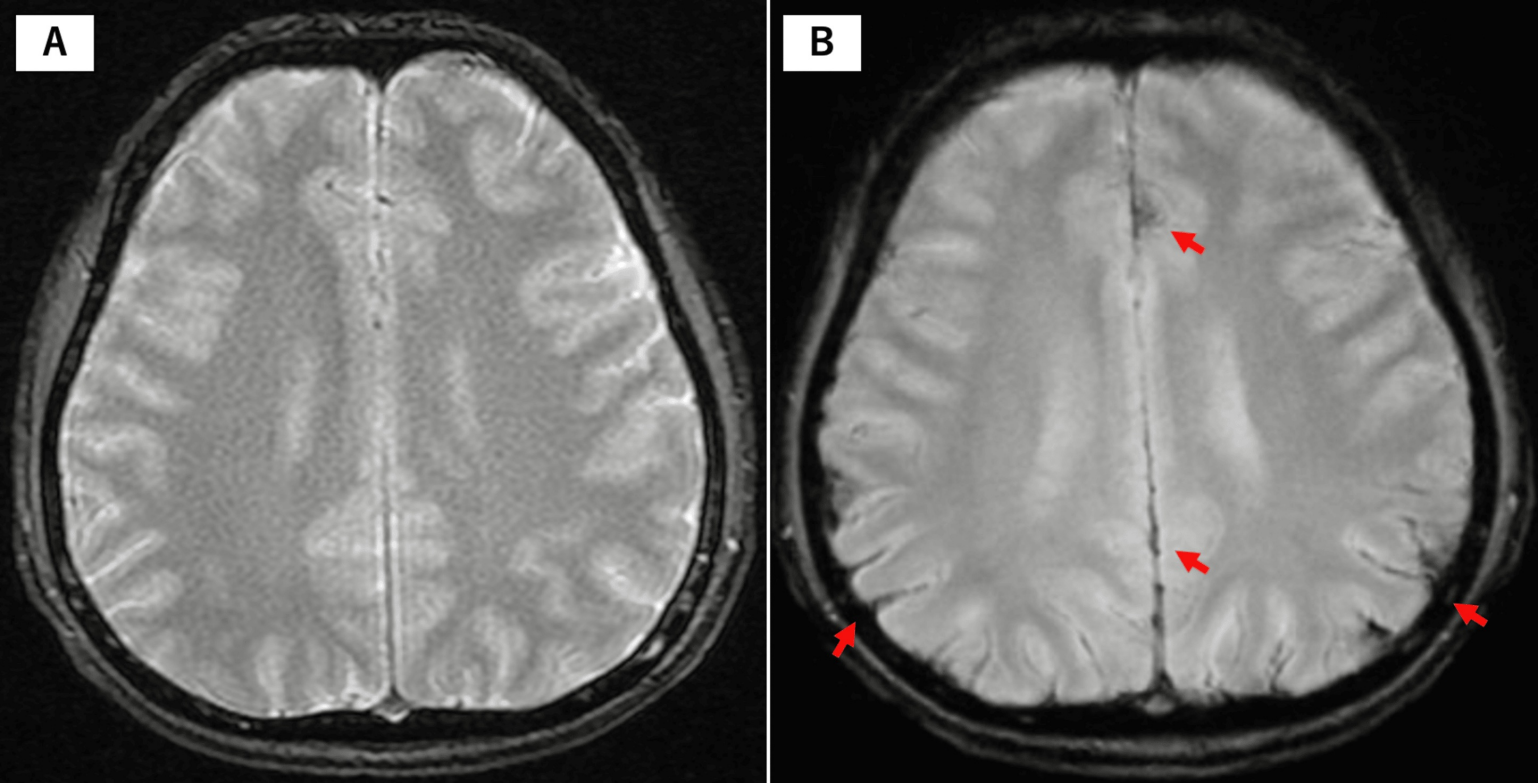
(A) Initial T2*-WI showed no apparent SAH intracranially. (B) However, upon admission to our hospital, a small amount of SAH was detected in both cerebral hemispheres. T2*-WI: T2*-weighted imaging; SAH: Subarachnoid hemorrhage.

Notably, there was no evidence of SAH in the intracranial space (Figure 2, Panel A). Based on initial MRI findings, the patient was diagnosed with a spinal subdural hematoma. However, the cause of the bleeding remained unclear, and the patient's headache gradually worsened. After suspicion of shunt disease at the craniocervical junction, the patient was transferred to our hospital for further evaluation. MRI T2*-weighted images revealed a small amount of SAH (Figure 2, Panel B).

**Figure 3:**
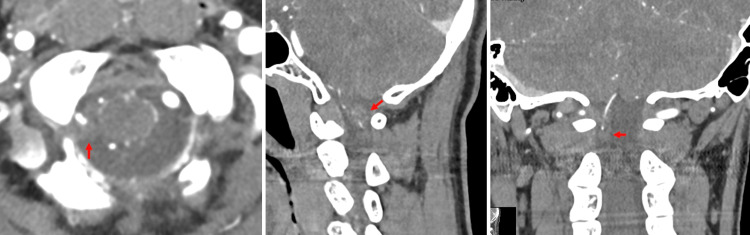
CT angiography showed no apparent abnormal vessels around the hematoma: (A) axial, (B) sagittal, and (C) coronal views. Red arrows indicate hematoma.

**Figure 4:**
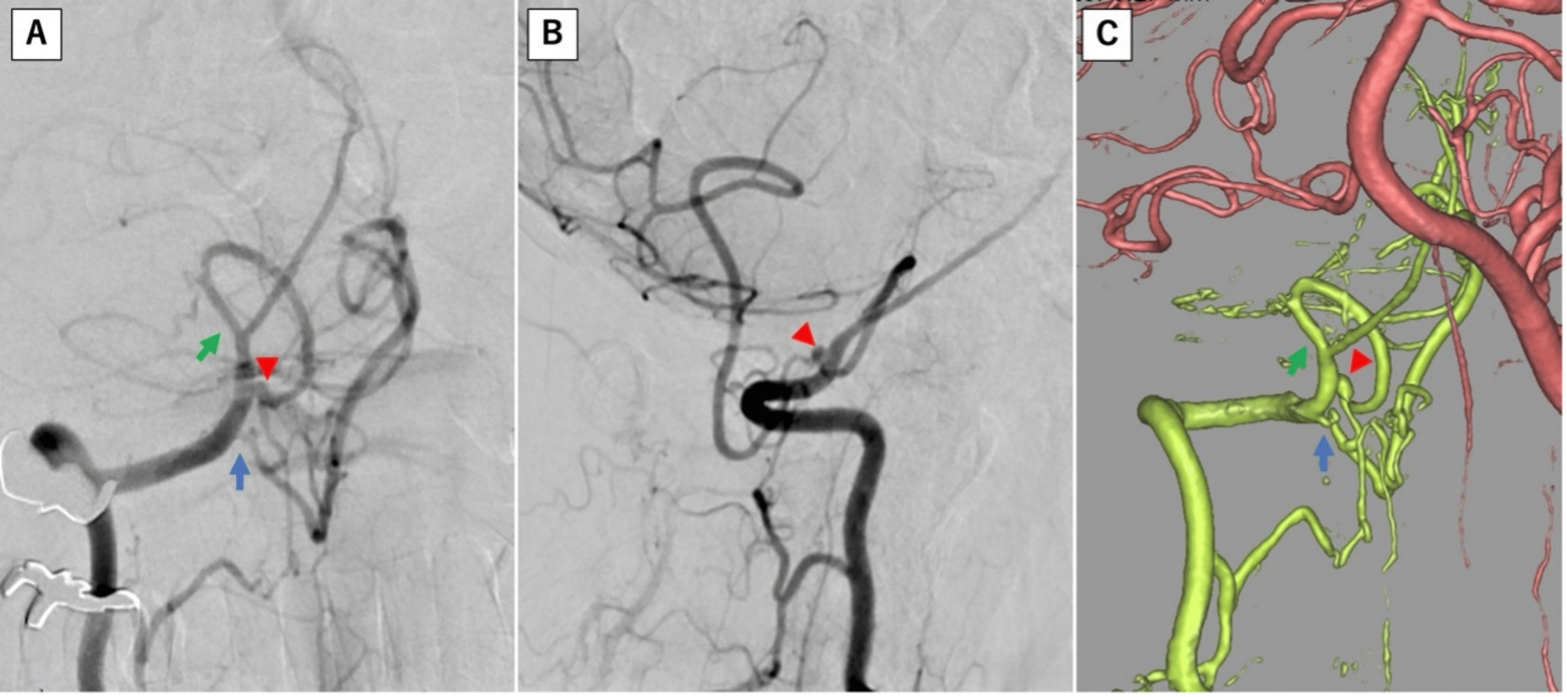
DSA of the right vertebral artery (A: anteroposterior view, B: lateral view, and C: 3D anteroposterior view). The right PICA demonstrates a double origin (blue and green arrows). A dissecting aneurysm (red triangle) is observed distal to the origin of the distal PICA (green arrow). Both origins converge distal to the dissected segment and continue as the vermian branch. DSA: Digital subtraction angiography; PICA: Posterior inferior cerebellar artery.

CT angiography was performed, but no apparent shunt vessels were identified around the hematoma (Figure 3). DSA was performed, revealing a double-origin right PICA. A dissecting aneurysm was identified in the peripheral segment of the distal origin, and both origins converge distal to the dissected segment and continue as the vermian branch (Figure 4).

**Figure 5:**
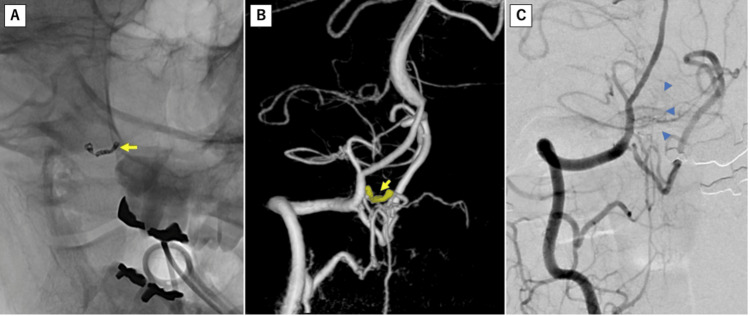
(A, B) Coil embolization (yellow arrow) was performed to trap the dissected segment via the distal side origin. Confirmation of the disappearance of the dissected segment was obtained (blue triangles, C).

This was determined to be the source of bleeding, and occlusion of the parent vessel was considered necessary. The patient underwent endovascular treatment with coil embolization for parent artery occlusion, a less invasive approach than direct surgical clipping. The procedure was performed under general anesthesia at the right inguinal fold. A guiding catheter (Chaperon 5F, Terumo Neuro, California) was placed in the right vertebral artery using a Radifocus 0.035 180 cm guidewire (Terumo Medical Corporation, NJ). Subsequently, 5000 IU of heparin was administered intravenously. An Excelsior SL-10 150cm microcatheter (Stryker Corporation, Kalamazoo, MI) was then navigated to the dissecting aneurysm via the origin of the distal side of the right PICA, using a Synchro SELECT SOFT guidewire (Stryker Corporation, Kalamazoo, MI). Coil embolization was performed using the following coils: two Target 360 NANO 1.5 mm x 30 mm (Stryker Corporation, Kalamazoo, MI), one Target 360 NANO 1 mm x 30 mm, and one Target 360 NANO 1 mm x 20 mm. A right vertebral artery angiogram was performed to confirm the occlusion of the dissecting aneurysm (Figure 5). It was observed that out of the two right PICAs, the proximal PICA was preserved, and residual blood flow to the peripheral vermian branch was maintained. Cilostazol administration was initiated postoperatively.

MRI performed on the day after the procedure revealed no evidence of ischemic lesions, and no other complications were observed. Persistent headaches suggested intracranial hypertension. Lumbar puncture revealed an opening pressure exceeding 350 mmH_2_O, prompting 25 ml of cerebrospinal fluid drainage. Due to headache recurrence shortly after, a lumbar drain was inserted for intracranial pressure management. Following symptomatic improvement and decreased cerebrospinal fluid output, the drain was removed after five days.

A follow-up DSA performed one week after the procedure demonstrated no signs of vasospasm and persistent occlusion of the embolized PICA. The patient was discharged 14 days after admission without any neurological deficits. At the seven-month outpatient follow-up, the patient showed no residual symptoms, and the MRI revealed no recurrence or new abnormalities.

## Discussion

This case report highlights a significant finding in the context of a PICA-dissecting aneurysm. The atypical presentation of a ruptured PICA-dissecting aneurysm with a hematoma localized to the craniocervical junction and upper cervical cord without an apparent intracranial subarachnoid hemorrhage has led to diagnostic challenges and delayed treatment. Despite the initial diagnostic delay, we were able to make the correct diagnosis before the re-rupture occurred, and successful endovascular treatment resulted in a favorable outcome. This finding underscores the importance of considering PICA-dissecting aneurysms in the differential diagnosis of isolated craniocervical junction hemorrhages, even in the absence of typical intracranial SAH. The atypical presentation in this case presented significant diagnostic challenges. Hematomas at the craniocervical junction can have various etiologies, including arterial dissection, arterial aneurysm rupture, dural arteriovenous fistula, arteriovenous malformation, trauma, tumors, and idiopathic causes [9-13]. Recent studies have also identified SARS-CoV-2 infection as a potential cause of spontaneous spinal hemorrhage, adding to the list of possible etiologies for spinal hematomas [14]. In the present case, the absence of an intracranial SAH at the time of onset made a dissecting aneurysm an unlikely initial consideration. This presentation differs markedly from the typical manifestation of distal PICA aneurysms, which usually present with an SAH or fourth ventricular hematoma [15]. While there have been reported cases of spinal hematoma associated with vertebral artery dissection [6,16], to our knowledge, there have been no previous reports of spinal hematoma caused by a dissecting aneurysm of the PICA. Interestingly, the patient's right PICA had a double origin, which may have contributed to the development of the dissection. This anatomical variation is associated with an increased risk of dissecting aneurysms [17]. A double-origin PICA is considered a remnant of the anastomosis between the PICA and lateral spinal artery [18].

**Figure 6:**
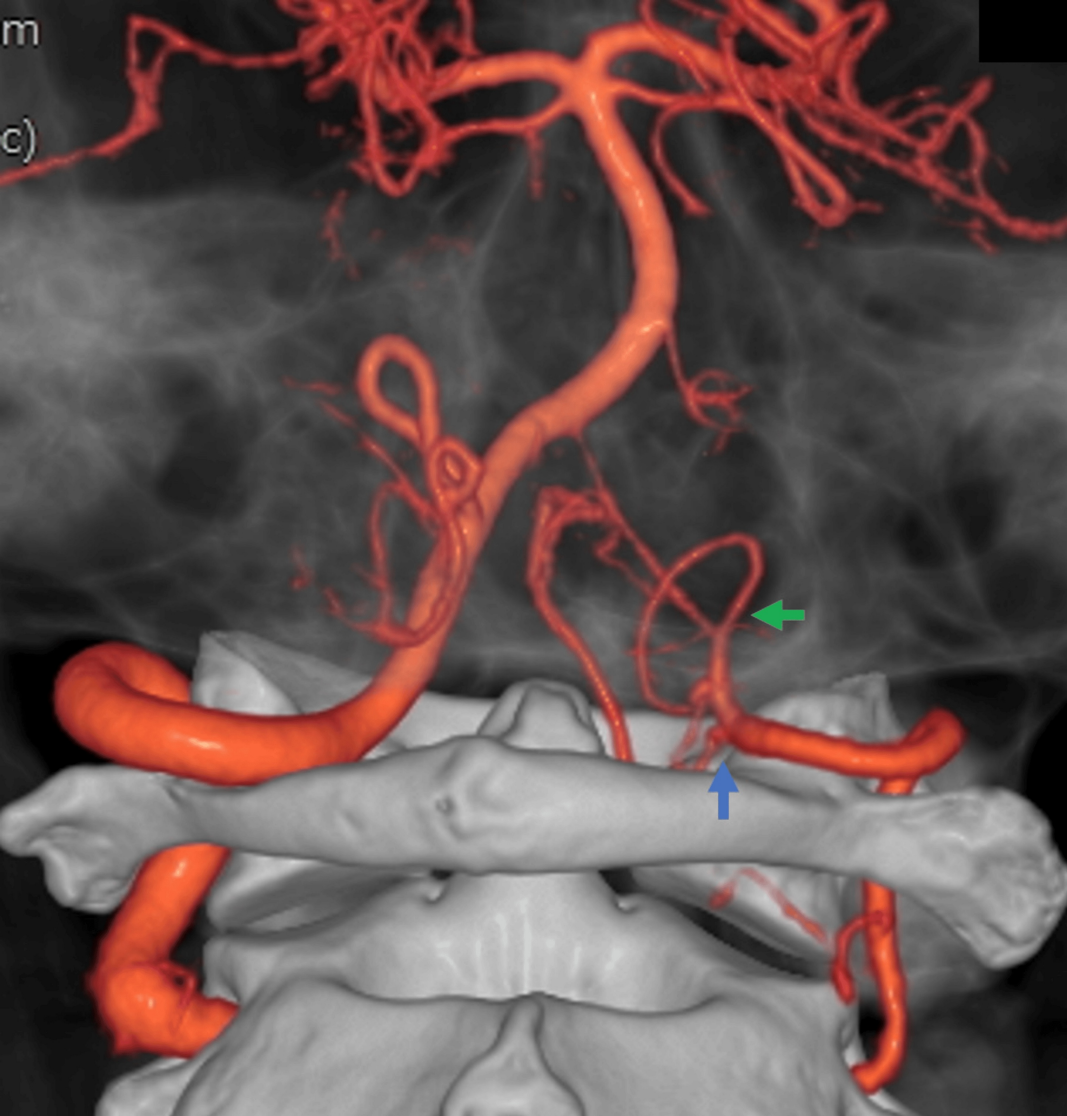
3D CT angiography revealed the proximal origin (blue arrow) arising from the distal part of the V3 segment and the distal origin (green arrow) emerging from the proximal part of the V4 segment

According to previous reports, the proximal origin of the double-origin PICA branches from the vertebral artery at the level of the C1/2-atlantooccipital, while the distal origin branches intracranially, with an average distance of 24.9 mm between the origins [19]. Considering that a normal PICA typically branches from the distal part of the V4 segment of the vertebral artery or vertebrobasilar junction [20], a double-origin PICA may run more caudally than a normal PICA. In this case, the proximal origin branched from the distal part of the V3 segment, while the distal origin branched from the proximal part of the V4 segment. This suggests that, due to the unique course of the double-origin PICA, the hematoma may have been localized to the craniocervical junction and cervical spine rather than the intracranial region. In this case, both origins of the double-origin PICA branched significantly more caudally than the typical PICA, with the proximal origin from the distal part of the V3 segment and the distal origin from the proximal part of the V4 segment (Figure 6). This markedly caudal course of the PICA, combined with the limited amount of hemorrhage, suggests that the hematoma resulting from the dissecting aneurysm may have been localized to the craniocervical junction and cervical spine rather than intracranially. However, further research is needed to validate this hypothesis.

Unlike vertebral artery dissections, which often have a more severe clinical course, previous studies have reported that many cases of ruptured distal PICA-dissecting aneurysms have favorable outcomes [21,22]. This may be related to the smaller vessel caliber and potentially slower blood flow in the distal PICA segments, contributing to a more contained hemorrhage, as observed in our case. However, these reports typically included only correctly diagnosed cases. There may be instances where delayed diagnosis or misdiagnosis leads to re-rupture and potentially severe consequences [23].

This case underscores the importance of a comprehensive and dynamic approach to diagnosing atypical vascular abnormalities. Initially, the patient presented with a headache; however, imaging revealed a hematoma at the craniocervical junction without apparent intracranial SAH. A follow-up CT scan later showed a small amount of SAH, likely due to the migration of blood from the craniocervical junction. This temporal evolution of the imaging findings highlights the critical importance of serial imaging in such cases. However, it is crucial to note that headaches and SAH can also occur in conditions without vascular abnormalities, such as spontaneous spinal subdural hematoma [24,25]. Therefore, while observing temporal changes, a definitive diagnosis of vascular lesions such as PICA-dissecting aneurysms ultimately requires angiography. This case emphasizes that in patients presenting with isolated craniocervical junction hemorrhage, especially when accompanied by headache, a high index of suspicion for vascular pathology should be maintained, and angiographic evaluation should be considered even in the absence of typical intracranial SAH.

One notable limitation of this case report is the absence of spinal DSA. Given the atypical distribution of the hematoma for a PICA-dissecting aneurysm, it would have been prudent to perform spinal DSA to definitively rule out the presence of any shunt vessels, even in the absence of abnormal vasculature on CT angiography. This additional imaging could have provided a more comprehensive vascular assessment and potentially yielded valuable insights into the etiology of the hematoma.

## Conclusions

This case highlights the importance of considering PICA-dissecting aneurysms in the differential diagnosis of isolated craniocervical junction hemorrhage, even in the absence of typical intracranial SAH. This atypical presentation leads to diagnostic challenges, emphasizing the need for a high index of suspicion and comprehensive vascular imaging, including angiography.
